# Designing a behaviour change intervention to address the behavioural risk factors for stillbirth: A study protocol

**DOI:** 10.12688/hrbopenres.13751.1

**Published:** 2023-07-26

**Authors:** Tamara Escañuela Sánchez, Karen Matvienko-Sikar, Richard A. Greene, Molly Byrne, Keelin O'Donoghue

**Affiliations:** 1 National Perinatal Epidemiology Centre, Department of Obstetric and Gynaecology, University College Cork, Cork, County Cork, Ireland; 2 Pregnancy Loss Research Group, Department of Obstetric and Gynaecology, University College Cork, Cork, County Cork, Ireland; 3 School of Public Health, University College Cork, Cork, Ireland; 4 Health Behaviour Change Research Group, School of Psychology, University of Galway, Galway, Galway, Ireland; 5 Infant Centre, University College Cork, Cork, County Cork, Ireland

**Keywords:** stillbirth, stillbirth prevention, modifiable risk factors, behaviour change intervention, COM-B model, BCW, evidence-based approach

## Abstract

**Introduction**

Stillbirth is a devastating outcome that, in some cases, has the potential to be prevented by addressing some of its modifiable risk factors such as smoking, alcohol consumption, illicit drug use, high maternal weight, lack of attendance to antenatal care, and sleep position. The aim of this study will be to design a behaviour change intervention focusing on the behavioural risk factors for stillbirth and based on the COM-B model and the Behaviour Change Wheel (BCW) systematic framework.

**Methods**

The first stage of the BCW framework involves understanding the target behaviour and defining the problem in behavioural terms. The second stage involves identifying intervention options, including intervention functions and policy categories. Finally, the third stage involves identifying content and implementation options, including behaviour change techniques (BCTs) and the mode of delivery. We will use multiple studies already conducted in our research team to inform the different stages of the BCW framework, these include a series of systematic reviews of the literature, qualitative interviews with pregnant women, and a survey study with healthcare professionals. Further, we will utilise a stakeholder group to obtain input through the process of the design of the intervention.

**Discussion**

This protocol provides a systematic and evidence-based approach to intervention design. The systematic review of the literature, qualitative interviews, and expert consensus workshops will ensure that the intervention design is based on the needs and preferences of pregnant women, healthcare professionals, and stakeholders involved in stillbirth prevention. The proposed intervention could be adapted and implemented in other settings to prevent stillbirth in high-risk populations.

## Introduction

Stillbirth is one of the most devastating outcomes that a woman and her family can experience ^1^. Although not all, some cases of stillbirth are preventable, and hence, efforts are being conducted internationally to tackle the risk factors for stillbirth in order to reduce its rates ^2^. Some of the modifiable risk factors for stillbirth include a behavioural component, meaning that they have the potential to be modified through behaviour change interventions. These risk factors are substance use (smoking ^3– 6^, alcohol consumption ^7, 8^, illicit drug use ^6^), high maternal weight ^9– 11^, lack of attendance to antenatal care ^12^, and sleep position ^13, 14^. Although some interventions tackling the behavioural risk factors for stillbirth already exist ^15– 17^, none of them have been designed to take into consideration all the different behavioural risk factors and the particularities regarding risk perception in the context of pregnancy and stillbirth, and with a behaviour change theory basis.

Behavioural theories are the accumulated knowledge of the assumptions of what behaviour is, how is it influenced, and what mechanisms of action will produce a change in behaviour ^18^. Behavioural theories are important because they can help explain and facilitate understanding of the factors that influence behaviour, and through which mechanisms this behaviour is influenced ^19^. Despite the importance and usefulness of behavioural theory, many behaviour change interventions have been designed without evidence or with a poor application of theory ^20^. Mitchie *et al.* (2011) hypothesise that this is because the existing frameworks to date did not meet intervention designer’s needs, as they either contained poorly defined constructs or did not produce enough level of detail to address what is effective in an intervention and what is not ^20^.

The COM-B model is a framework for understanding behaviour and how to change behaviour ^21^. This model was generated to develop a behaviour change design methodology informed by behaviour theory. The authors identified three main factors that are considered sufficient and necessary for behaviour change: the necessary skills, a strong intention to perform the behaviour, and no environmental constraints to make the behaviour possible ^18^. These are operationalised as Capability, Motivation, and Opportunity respectively. In the COM-B model, the three components interact and influence each other, hence, altering one has the potential to alter the others. In the COM-B model, Capability is defined as “the individual’s psychological and physical capacity to engage in the behaviour” ^18^ through, for example, knowledge or skills; and it is divided into Physical Capability and Psychological Capability. Opportunity is defined as “all the factors that lie outside the individual that make the behaviour possible or prompt it” ^18^; and it is subdivided into social opportunity and physical opportunity. Finally, Motivation is defined as “all those brain processes that energise and direct behaviour” ^18^ such as habitual processes, emotional responses, and analytical decision-making; and it is subdivided into reflexive motivation and automatic motivation.

The Theoretical Domains Framework (TDF) and the COM-B model represent potentially useful theories of behaviour that can be used in the context of stillbirth prevention because they incorporate internal factors, such as attitudes, co-occurring behaviours and co-founding factors ^22^. The importance of the social context has been described in the literature in relation to behaviour change during pregnancy ^23^. Moreover, it is also well established that the behaviours that have been associated with an increased risk of stillbirth often can co-occur ( *e.g.*, smoking and alcohol consumption, smoking and illicit drug use, illicit drug use and lack of attendance at antenatal care, substance use and physical inactivity) ^6, 24, 25^. Therefore, utilising a theory that takes into account factors that influence behaviour such as the physical and social environment is important to understand behaviour change during pregnancy ^26^. The COM-B model has not been widely tested in the context of stillbirth prevention. However, previous research mapping factors influencing dietary behaviour, physical activity, smoking, and alcohol use ^23^ during pregnancy to the elements of the COM-B has concluded that all its factors (capability, opportunity and motivation) have a role in directing behaviour ^26^.

The Behaviour Change Wheel (BCW) is a systematic framework providing a methodology for designing behaviour change interventions. This methodology proposes the elements of the COM-B model as mechanisms of action. The BCW provides intervention designers with a systematic process for intervention design composed of three main stages and eight steps exposed in Table 1.

**Table 1.** Stages and steps composing the Behaviour Change Wheel systematic process for intervention design.

Stage 1: Understanding the target behaviour	Step 1: define the problem in behavioural terms.
	Step 2: Select the target behaviour.
	Step 3: Specify the target behaviour.
	Step 4: identify what needs to change.
Stage 2: Identify intervention options	Step 5: Identify intervention functions.
	Step 6: Identify policy categories.
Stage 3: Identify content and implementation options	Step 7: Identify behaviour change techniques (BCTs)
	Step 8: Identify the mode of delivery.

The BCW framework has been used for behaviour change intervention design in the context of pregnancy. For example, Gould *et al.* (2017) successfully used the BCW to design an intervention to target smoking amongst Australian indigenous pregnant women ^27^. Another example of an intervention designed using the BCT is the “stay-active” smartphone app developed by Smith *et al.* (2022) to increase physical activity in pregnant women ^28^. The authors of both interventions report that utilising the BCW provided them with a systematic approach that facilitated the process. The process described in the studies published by both authors outlining their use of the BCW to inform the development of their interventions led to the design of two different interventions that are now being tested through feasibility studies ^29, 30^. Hence, there is evidence in the literature that the BCW is a methodology suitable for intervention design applicable to pregnancy.

While the BCW provides a promising approach to informing design of behaviour change interventions that target behaviours associated with increased risk of stillbirth, to date, no intervention has been designed using the BCW and adopting a multi-target approach that might incorporate all of the relevant behaviours. Evidence from public health initiatives implemented in high-income countries provide evidence that further reduction in stillbirth rates is possible in Ireland ^31, 32^. Hence, the main objective of this project is to utilise all of the evidence gathered in earlier stages of its development ^33– 39^ to inform the design of a behaviour change intervention tackling the modifiable risk factors for stillbirth.

## Methods

Using all of the evidence generated from the studies composing this thesis, we will follow the process proposed by the BCW for intervention design.

### Sources of evidence

The RELEVANT Study team is composed by researchers with expertise in different areas including health psychology, fetal and maternal medicine, population/public health, and behaviour change. A total of six projects composed of eight studies form the RELEVANT Study : (1) a literature review of risk factors for stillbirth; (2) a quantitative website content analysis exploring information provided online regarding stillbirth and risk factors; (3) three qualitative evidence syntheses exploring facilitators and barriers to modifying substance use, weight management, and antenatal care attendance; (4) a qualitative study exploring postpartum women’s experience of antenatal health education and their awareness of stillbirth and risk factors; (5) a systematic review of interventions to prevent stillbirth in high-income countries; and (6) a survey of healthcare professionals to identify barriers to communicating information about stillbirth and risk factors. The data obtained in these studies will be utilised to inform the BCW process for the development of a behaviour change intervention. For more information on the steps and stages of the Behaviour Change Wheel that each study will inform, please refer to Table 2.

**Table 2.** Sources of evidence, aims, findings and steps of the BCW informed.

Source code: Source title	Study design	Overview of aims	Overview of findings	Behaviour Change Wheel phase(s) and step(s) informed
S1: “Modifiable risk factors for stillbirth: a literature review” ^35^	Literature review	• To explore and examine the available evidence in relation to behavioural risk factors for stillbirth. • To define the problem and select target behaviours.	Four main modifiable risk factors with a behavioural component were found to have the strongest evidence: ▪ Substance use (smoking, alcohol, illicit drugs) ▪ Maternal weight ▪ Attendance & compliance with antenatal care. ▪ Sleep position.	Stage 1: Steps 1, 2
S2: “Stillbirth and risk factors: an evaluation of Irish and UK websites” ^37^	Quantitative content analysis	• To assess whether the current online resources for pregnant women are useful to obtain information about stillbirth and behavioural risk factors.	▪ <50% of websites contained information about stillbirth ▪ <30% of websites contained information about risk factors for stillbirth ▪ Only one website contained all the information sought about stillbirth ( *e.g.* definition, prevalence, *etc.*) & risk factors.	Stage 1: Step 4 Stage 3: Step 8
S3: “Facilitators and barriers to substance-free pregnancies in high-income countries: A meta-synthesis of qualitative research.” ^39^ “Facilitators and barriers to seeking and engaging with antenatal care in high- income countries: A meta- synthesis of qualitative research.” ^38^ “Facilitators and barriers influencing weight management behaviours during pregnancy: a meta- synthesis of qualitative research.” ^34^	Qualitative evidence synthesis	• To assess the literature in order to identify barriers and facilitators to women’s behaviour change regarding attendance and engaging with antenatal care, substance use, and weight management behaviours from the pregnant women’s perspective.	Identified areas of concern: ▪ Health literacy, awareness of risks & benefits ▪ Insufficient & overwhelming sources of information ▪ Lack of opportunities & HCPs' attitudes interfering with communication & discussion ▪ Social influence of the environment ▪ Social judgement, stigmatisation of women. *A search to identify facilitators and barriers influencing sleep position was also conducted at two different points in time during this PhD, however, no qualitative research was identified.	Stage 1: Steps 1, 2, 3, 4 Stage 2: Steps 5, 6 Stage 3: Steps 7, 8
“Exploring first time mothers’ experiences and knowledge about behavioural risk factors for stillbirth” ^33^	Qualitative Study	To explore women’s experiences of behaviour change during pregnancy & awareness regarding stillbirth and associated risk factors.	▪ Behaviour change during pregnancy perceived as easy and natural. ▪ Women had high level of awareness regarding health advice, but very limited regarding stillbirth. ▪ There is a lack of discussion with HCPs about stillbirth & risks, so women rely on their own information-seeking behaviours. ▪ Women had a general positive attitude towards receiving information about stillbirth; knowledge perceived as key.	Stage 1: Steps 1, 2, 3, 4 Stage 2: Steps 5, 6 Stage 3: Steps 7, 8
“A systematic review of behaviour change techniques used in the context of stillbirth prevention.” [Manuscript in preparation]	Systematic Review	To identify the behaviour change techniques used to date in stillbirth prevention interventions.	▪ 9 interventions were included in analysis. ▪ The most common BCT used was “Information about health consequences”, followed by “Adding objects to the environment”. ▪ The maximum number of BCTs was 11 and the minimum was 2.	Phase 1: Steps 3, 4 Phase 2: Steps 5, 6 Phase 3: Steps 7, 8
“Exploring healthcare professionals’ experiences when communicating with pregnant women about stillbirth.” [Manuscript in preparation]	Online survey study	To explore maternity healthcare professionals experience and knowledge regarding stillbirth and modifiable risk factors for stillbirth. To explore healthcare professionals’ common practices regarding information provision about stillbirth, risk factors and health advice. To explore common barriers to information provision about stillbirth, risk factors and health advice.	▪ Only 50% of the surveyed healthcare professionals (HCPs) correctly identified the Irish definition of stillbirth. ▪ Attendance at antenatal care was perceived as the most important risk factor to discuss with pregnant women, followed by smoking. ▪ Maternal weight was the risk factor that HCPs found most challenging to discuss with women, and pregnant women were perceived as being reluctant to discuss it with HCPs. ▪ Time constraints were identified as a major barrier to providing education and supporting behaviour change in pregnancy. ▪ While 65.2% of HCPs considered informing women about health behaviours and stillbirth risks as part of their role, only 56.4% felt confident and trained to do so. ▪ The study highlights the need for prioritizing HCP education and providing protected time to discuss modifiable risk factors during antenatal care to enhance stillbirth preventive efforts.	Phase 1: Step 3, 4 Phase 2: Step 5 Phase 3: Steps 7, 8

### Applying the Behaviour Change Wheel

***Stage 1: Identification of behavioural barriers and facilitators to modify the behavioural risk factors for stillbirth during pregnancy.*** Stage 1 is informed by the first phase of the BCW, which requires a deep examination and understanding of the relevant behaviour/s. This involves defining the behaviour in terms of who, what, where, when, and how often ^21^. In this case, the relevant behaviours are substance use, attendance and engaging with antenatal care, weight management behaviours (diet, physical activity) and sleep position. It is important that our approach takes into consideration all of the different behaviours, as interventions for these individual behaviours already exist. When it comes to behaviour change, our *who* are women, and *when* should be throughout pregnancy or the pre-conceptual period.

Following from the behavioural specifications of the behaviours, the facilitators and barriers identified in the studies will be extracted verbatim from the papers and coded using the components of the COM-B framework. The findings from this coding will then be synthesised narratively and using matrices and tables.

***Stage 2: Identification of behavioural intervention strategies to promote behaviour change during pregnancy.*** We will use the BCW framework to identify and select intervention functions. The intervention functions are mapped into the elements of the COM-B model. Hence, the findings of Stage 1 will inform Stage 2 by mapping the identified behavioural components of the COM-B model into intervention functions. If multiple intervention functions are identified as relevant, the APEASE criteria will be used to prioritise the selection of the most affordable, practical, effective, acceptable, safe and equitable (APEASE).

During the process of intervention design and the application of the APEASE criteria, we will include stakeholder groups throughout. The stakeholder group should then involve health care professionals (HCPs), women from different sociodemographic backgrounds, as well as women who have used the currently available supports for behaviour change during pregnancy. Healthcare professionals and patient representatives will be identified from existing professional networks through the members of our research group, and by contacting the relevant support associations or using social media. The meetings will not include more than 15 members and no less than 10, with recruitment being focus on diversity and making sure all relevant groups are represented. The meetings will be held online. Before each meeting, all participants will receive a lay summary of the advancements made to date in the project, to ensure that everybody has the same level of awareness as to what is required of them in that meeting. In these sessions, considerations around the implementation and anticipated effectiveness of each intervention function and BCT previously identified will be discussed. These discussions will result in a ranking of the different intervention functions and BCTs by perceived importance. The application of the APEASE criteria will be conducted by two or more investigators independently, and support and input from the steering group will be then sought.

After identifying intervention functions, the next step will be to identify potential intervention content in terms of BCTs. To identify BCTs, the BCTTv1 will be used. Then, the relevant BCTs will be operationalised by translating them into a concrete application. The APEASE criteria and stakeholder input will also be utilised in this stage to prioritise the selection of BCTs.

### Ethical considerations

The University College Cork (UCC) Code of Research conduct ethical approval and the General Data Protection Regulations (GDPR) procedures will be followed for all research activities. The first two stages do not involve any potential ethical concerns as they only involve utilising data obtain through the review of the literature or findings from studies that were granted ethical approval by the Cork Research Ethical Committee for the Cork Teaching Hospital in UCC when conducted.

For the creation and involvement of the stakeholder group, ethical approval will be sought from the same ethical committee. Potential participants will be provided with information leaflets making clear that participation is voluntary and that the meetings will be recorded for data collection. Written informed consent will be obtained to participate in the meetings. Participants will be informed that all data will be stored anonymously.

## Study status

At the moment, the research team is completing stage 1 of this study and coding the data into the COM-B model as described in the stage one of the “Applying the Behaviour Change Wheel” section.

## Conclusion

In summary, following this process will hopefully allow us to define the behaviours that need to be addressed, identify intervention functions, and identify BCTs. This process also will allow us to identify options to translate such BCTs into actual intervention content. Involving PPIs and stakeholders and utilising the APEASE criteria throughout the whole process will enhance the chances of the intervention to be acceptable and effective for its purpose. The following steps will then involve developing an implementation strategy for the designed intervention.

## Data Availability

No data are associated with this article.

## References

[1] Frøen JF Cacciatore J McClure EM : Stillbirths: Why they matter. *Lancet.* 2011;377(9774):1353–1366. 10.1016/S0140-6736(10)62232-5 21496915

[2] De Bernis L Kinney MV Stones W : Stillbirths: Ending preventable deaths by 2030. *Lancet.* 2016;387(10019):703–716. 10.1016/S0140-6736(15)00954-X 26794079

[3] Marufu TC Ahankari A Coleman T : Maternal smoking and the risk of still birth: systematic review and meta-analysis. *BMC Public Health.* 2015;15: 239. 10.1186/s12889-015-1552-5 25885887 PMC4372174

[4] Flenady V Koopmans L Middleton P : Major risk factors for stillbirth in high-income countries: a systematic review and meta-analysis. *Lancet.* 2011;377(9774):1331–1340. 10.1016/S0140-6736(10)62233-7 21496916

[5] Odendaal H Dukes KA Elliott AJ : Association of Prenatal Exposure to Maternal Drinking and Smoking with the Risk of Stillbirth. *JAMA Netw Open.* 2021;4(8): e2121726. 10.1001/jamanetworkopen.2021.21726 34424306 PMC8383134

[6] Varner MW Silver RM Hogue CJR : Association between stillbirth and illicit drug use and smoking during pregnancy. *Obstet Gynecol.* 2014;123(1):113–125. 10.1097/AOG.0000000000000052 24463671 PMC3931517

[7] Andersen AMN Andersen PK Olsen J : Moderate alcohol intake during pregnancy and risk of fetal death. *Int J Epidemiol.* 2012;41(2):405–413. 10.1093/ije/dyr189 22253313

[8] Cornman-Homonoff J Kuehn D Aros S : Heavy prenatal alcohol exposure and risk of stillbirth and preterm delivery. *J Matern Fetal Neonatal Med.* 2012;25(6):860–863. 10.3109/14767058.2011.587559 21728738 PMC4148070

[9] Chu SY Kim SY Lau J : Maternal obesity and risk of stillbirth: a metaanalysis. *Am J Obstet Gynecol.* 2007;197(3):223–228. 10.1016/j.ajog.2007.03.027 17826400

[10] Yao R Goetzinger K Caughey A : Obesity and stillbirth: is there a dose-response effect?2018. 10.1016/j.ajog.2017.11.292

[11] Yao R Ananth CV Park BY : Obesity and the risk of stillbirth: a population-based cohort study. *Am J Obstet Gynecol.* 2014;10(5): 457.e1–457.e9. 10.1016/j.ajog.2014.01.044 24674712

[12] Stacey T Thompson JMD Mitchell EA : Antenatal care, identification of suboptimal fetal growth and risk of late stillbirth: Findings from the Auckland Stillbirth Study. *Aust N Z J Obstet Gynaecol.* 2012;52(3):242–247. 10.1111/j.1479-828X.2011.01406.x 22276935

[13] Stacey T Thompson JMD Mitchell EA : The Auckland stillbirth study, a case-control study exploring modifiable risk factors for third trimester stillbirth: Methods and rationale. *Aust N Z J Obstet Gynaecol.* 2011;51(1):3–8. 10.1111/j.1479-828X.2010.01254.x 21299501

[14] O’Brien LM Warland J Stacey T : Maternal sleep practices and stillbirth: Findings from an international case-control study. *Birth.* 2019;46(2):344–354. 10.1111/birt.12416 30656734 PMC7379524

[15] Chamberlain C O'Mara-Eves A Porter J : Psychosocial interventions for supporting women to stop smoking in pregnancy. *Cochrane Database Syst Rev.* 2017;2(2): CD001055. 10.1002/14651858.CD001055.pub5 28196405 PMC6472671

[16] Gilinsky A Swanson V Power K : Interventions delivered during antenatal care to reduce alcohol consumption during pregnancy: A systematic review. *Addict Res Ther.* 2011;19(3):235–250. 10.3109/16066359.2010.507894

[17] Currie S Sinclair M Murphy MH : Reducing the Decline in Physical Activity during Pregnancy: A Systematic Review of Behaviour Change Interventions. *PLoS One.* 2013;8(6): e66385. 10.1371/journal.pone.0066385 23799096 PMC3682976

[18] Davis R Campbell R Hildon Z : Theories of behaviour and behaviour change across the social and behavioural sciences: a scoping review. *Health Psychol Rev.* 2015;9(3):323–344. 10.1080/17437199.2014.941722 25104107 PMC4566873

[19] Patey AM Hurt CS Grimshaw JM : Changing behaviour ’more or less’-do theories of behaviour inform strategies for implementation and de-implementation? A critical interpretive synthesis. *Implement Sci.* 2018;13(1): 134. 10.1186/s13012-018-0826-6 30373635 PMC6206907

[20] Michie S van Stralen M West R : The behaviour change wheel: A new method for characterising and designing behaviour change interventions. *Implement Sci.* 2011;6: 42. 10.1186/1748-5908-6-42 21513547 PMC3096582

[21] Michie S Atkins L West R : The Behaviour Change Wheel: A Guide to Designing Interventions. (Silverback Publishing.,) 2014;329. Reference Source

[22] Atkins L Francis J Islam R : A guide to using the Theoretical Domains Framework of behaviour change to investigate implementation problems. *Implement Sci.* 2017;12(1): 77. 10.1186/s13012-017-0605-9 28637486 PMC5480145

[23] Rockliffe L Peters S Heazell AEP : Factors influencing health behaviour change during pregnancy: a systematic review and meta-synthesis. *Health Psychol Rev.* 2021;15(4):613–632. 10.1080/17437199.2021.1938632 34092185

[24] Lange S Probst C Quere M : Alcohol use, smoking and their co-occurrence during pregnancy among Canadian women, 2003 to 2011/12. *Addict Behav.* 2015;50:102–9. 10.1016/j.addbeh.2015.06.018 26117214

[25] Meader N King K Moe-Byrne T : A systematic review on the clustering and co-occurrence of multiple risk behaviours. *BMC Public Health.* 2016;16: 57. 10.1186/s12889-016-3373-6 27473458 PMC4966774

[26] Rockliffe L Peters S Heazell AEP : Understanding pregnancy as a teachable moment for behaviour change: a comparison of the COM-B and teachable moments models. *Health Psychol Behav Med.* 2022;10(1):41–59. 10.1080/21642850.2021.2014851 34993005 PMC8725882

[27] Gould GS Bar-Zeev Y Bovill M : Designing an implementation intervention with the Behaviour Change Wheel for health provider smoking cessation care for Australian Indigenous pregnant women. *Implement Sci.* 2017;12(1): 114. 10.1186/s13012-017-0645-1 28915815 PMC5602934

[28] Smith R Michalopoulou M Reid H : Applying the behaviour change wheel to develop a smartphone application ‘stay-active’ to increase physical activity in women with gestational diabetes. *BMC Pregnancy Childbirth.* 2022;22(1): 253. 10.1186/s12884-022-04539-9 35346075 PMC8962081

[29] Smith R Kenworthy Y Astbury NM : Study protocol: use of a smartphone application to support the implementation of a complex physical activity intervention (+ *Stay Active*) in women with gestational diabetes mellitus-protocol for a non-randomised feasibility study. *BMJ Open.* 2022;12(9): e062525. 10.1136/bmjopen-2022-062525 36171028 PMC9528591

[30] Template P : Date and version No: 20/02/2014, Version 1.2014;1–22.

[31] Andrews CJ Ellwood D Middleton PF : Implementation and evaluation of a quality improvement initiative to reduce late gestation stillbirths in Australia: Safer Baby Bundle study protocol. *BMC Pregnancy Childbirth.* 2020;20(1): 694. 10.1186/s12884-020-03401-0 33187483 PMC7664588

[32] NHS England: Saving Babies’ Lives Version Two. 2019. Reference Source

[33] Escañuela Sánchez T Matvienko‐Sikar K Meaney S : Exploring first time mothers’ experiences and knowledge about behavioural risk factors for stillbirth. *Health Expect.* 2022;26(1):329–342. 10.1111/hex.13662 36416378 PMC9854314

[34] Escañuela Sánchez T Meaney S O'Connor C : Facilitators and barriers influencing weight management behaviours during pregnancy: a meta-synthesis of qualitative research. *BMC Pregnancy Childbirth.* 2022;22(1): 682. 10.1186/s12884-022-04929-z 36064379 PMC9443069

[35] Escañuela Sánchez T Meaney S O’Donoghue K : Modifiable risk factors for stillbirth: a literature review. *Midwifery.* 2019;79: 102539. 10.1016/j.midw.2019.102539 31585399

[36] Escañuela Sánchez T Byrne M Meaney S : A protocol for a systematic review of behaviour change techniques used in the context of stillbirth prevention [version 1 ; peer review : 1 approved with reservations]. * HRB Open Res.* 2021;4: 92. 10.12688/hrbopenres.13375.2 36743684 PMC9874168

[37] Escañuela Sánchez T Meaney S O’Donoghue K : Stillbirth and risk factors : an evaluation of Irish and UK websites. *J Commun Healthc.* 2020;14(1):68–77. 10.1080/17538068.2020.1807887

[38] Escañuela Sánchez T Linehan L O’Donoghue K : Facilitators and barriers to seeking and engaging with antenatal care in high‐income countries: A meta‐synthesis of qualitative research. *Health Soc Care Community.* 2022;30(6):e3810–e3828. 10.1111/hsc.14072 36240064 PMC10092326

[39] Escañuela Sánchez T Matvienko-Sikar K Linehan L : Facilitators and barriers to substance-free pregnancies in high-income countries: A meta-synthesis of qualitative research. *Women Birth.* 2022;35(2):e99–e110. 10.1016/j.wombi.2021.04.010 33935004

