# Supplementary material for: Designing a behaviour change intervention to address the behavioural risk factors for stillbirth: A study protocol
Source: HRB Open Res. 2023 Jul 26;6:35. [Version 1] doi: 10.12688/hrbopenres.13751.1 (PMC11803438; doi:10.12688/hrbopenres.13751.1)
Supplement: Peer review history

# Reviewer report: Reviewer response for version 1

**DOI:** 10.21956/hrbopenres.15042.r43571

**Published:** 2025-01-02

**Authors:** Sarah Saleem

**Affiliations:** 1 Aga Khan University, Karachi, Pakistan

This is a study protocol whichis related to development of an intervention to change behaviour in relation to prevention of Stillbirths. The authors have identified modifiable risk facors through eight studies and developing an intervention in an holistic manner using COM-b model and behaviour change wheel systematic framework. The study will also involve stakeholders to obtain input through the process of the design of the intervention. The interventions will be implemented in high risk populations

 Very clearly written protocol, which is addressing an important aspect of stillbirth occurrence.

Is the study design appropriate for the research question?

Yes

Is the rationale for, and objectives of, the study clearly described?

Yes

Are sufficient details of the methods provided to allow replication by others?

Yes

Are the datasets clearly presented in a useable and accessible format?

Yes

Reviewer Expertise:

medical Doctor, Epidemiology, maternal and Newborn health, stillbirths, family planning

I confirm that I have read this submission and believe that I have an appropriate level of expertise to confirm that it is of an acceptable scientific standard.

# Reviewer report: Reviewer response for version 1

**DOI:** 10.21956/hrbopenres.15042.r43570

**Published:** 2024-12-12

**Authors:** Philippa Middleton

**Affiliations:** 1 Women and Kids, SAHMRI and The University of Adelaide, Adelaide, South Australia, Australia

Abstract:

 Methods: I'm not sure that 'mode of delivery' fits here. 

 Methods: Line 4: suggest deleting 'multiple" here

 Introduction:

 Line 1: suggest deleting "Although not all"..

 Para 2: change 'Mitchie" to "Michie"

 Para 3: rephrase "no environmental constraints..."

 Para 3: suggest adding some more clarification about reflexive/automatic motivation (or omitting here and adding it to main text instead).

 As parts of the manuscript seem very similar to the authors' 2021 paper (ref 36), I suggest removing any unnecessary duplication.

 I also wonder if Table 1 is necessary.

 ref 30: this does not look complete; also the next sentence does not seem necessary.

 Last paragraph in the introduction is not needed.  

 Refs 33-39 are all self-cites - suggest some, if not all, be removed

 APEASE is not defined or explained.

Is the study design appropriate for the research question?

Yes

Is the rationale for, and objectives of, the study clearly described?

Partly

Are sufficient details of the methods provided to allow replication by others?

Partly

Are the datasets clearly presented in a useable and accessible format?

Not applicable

Reviewer Expertise:

stillbirth, preterm birth, nutrition, implementation, guidelines

I confirm that I have read this submission and believe that I have an appropriate level of expertise to confirm that it is of an acceptable scientific standard, however I have significant reservations, as outlined above.

# Reviewer report: Reviewer response for version 1

**DOI:** 10.21956/hrbopenres.15042.r36309

**Published:** 2023-10-11

**Authors:** Tomasina Stacey

**Affiliations:** 1 King's College London, Florence Nightingale Faculty of Nursing Midwifery and Palliative Care, London, UK

This paper by Sanchez et al sets out the protocol for an intervention to address behavioural risk factors for stillbirth. The proposed development of an intervention to promote behaviour change  builds on the excellent work already conducted by the study team. Clear rationale and justification for the research is provided. They have used the COM-B model to inform the framing of their intervention – this provides a useful theoretical grounding.

 The only concern that I have is that the behaviours identified (smoking, alcohol consumption, illicit drug use, high maternal weight, antenatal care attendance and sleep position) are very diverse and are likely to require different strategies to address them, they also may require the targeting of specific (and varying) population groups. There is some strength in combining these factors, but also a risk that by doing so none will be addressed adequately- although this issue is touched upon, it is not fully explored in this short paper.

Is the study design appropriate for the research question?

Yes

Is the rationale for, and objectives of, the study clearly described?

Yes

Are sufficient details of the methods provided to allow replication by others?

Yes

Are the datasets clearly presented in a useable and accessible format?

Not applicable

Reviewer Expertise:

Modifiable risk for stillbirth

I confirm that I have read this submission and believe that I have an appropriate level of expertise to confirm that it is of an acceptable scientific standard.
